# AromaDb: A Database of Medicinal and Aromatic Plant’s Aroma Molecules With Phytochemistry and Therapeutic Potentials

**DOI:** 10.3389/fpls.2018.01081

**Published:** 2018-08-13

**Authors:** Yogesh Kumar, Om Prakash, Himanshu Tripathi, Sudeep Tandon, Madan M. Gupta, Laiq-Ur Rahman, Raj K. Lal, Manoj Semwal, Mahendra Pandurang Darokar, Feroz Khan

**Affiliations:** ^1^Department of Metabolic and Structural Biology, CSIR-Central Institute of Medicinal and Aromatic Plants, Lucknow, India; ^2^Department of Process Chemistry and Chemical Engineering, CSIR-Central Institute of Medicinal and Aromatic Plants, Lucknow, India; ^3^Department of Analytical Chemistry, CSIR-Central Institute of Medicinal and Aromatic Plants, Lucknow, India; ^4^Department of Plant Biotechnology, CSIR-Central Institute of Medicinal and Aromatic Plants, Lucknow, India; ^5^Department of Plant Breeding, CSIR-Central Institute of Medicinal and Aromatic Plants, Lucknow, India; ^6^Information and Communication Technology Lab, CSIR-Central Institute of Medicinal and Aromatic Plants, Lucknow, India; ^7^Department of Molecular Bioprospection, CSIR-Central Institute of Medicinal and Aromatic Plants, Lucknow, India; ^8^Skaggs School of Pharmacy and Pharmaceutical Sciences, University of California, San Diego, San Diego, CA, United States

**Keywords:** medicinal, aromatic, plants, essential oils, chemotypes, aroma, molecule, bioactivity

## Abstract

In traditional, herbal medicine, and aromatherapy, use of essential oils and their aroma compounds have been known since long, for the management of various human diseases. The essential oil is a mixture of highly complex, naturally occurring volatile aroma compounds synthesized by medicinal and aromatic plants as secondary metabolites. Essential oils widely used in pharmaceutical, cosmetic, sanitary, food industry and agriculture for their antibacterial, antiviral, antifungal, antiparasitic, insecticidal, anticancer, neuroprotective, psychophysiological, and anti-aging activities. Moreover, volatile aroma compounds comprise a chemically diverse class of low molecular weight organic compounds with significant vapor pressure. However, aroma compounds produced by plants, mainly attract pollinators, seed dispersers and provide defense against pests or pathogens. However, in humans, about 300 active olfactory receptor genes are involved to detect thousands of different aroma compounds and modulates expression of different metabolic genes regulating human psychophysiological activity, brain function, pharmacological signaling, and therapeutic potential. Keeping in mind this importance, present database, namely, AromaDb (http://bioinfo.cimap.res.in/aromadb/) covers information of plant varieties/chemotypes, essential oils, chemical constituents, GC-MS profile, yield variations due to agro-morphological parameters, trade data, aroma compounds, fragrance type, and bioactivity details. The database includes 1,321 aroma chemical structures, bioactivities of essential oil/aroma compounds, 357 fragrance type, 166 commercially used plants, and their high yielding 148 varieties/chemotypes. Also includes calculated cheminformatics properties related to identification, physico-chemical properties, pharmacokinetics, toxicological, and ecological information. Also comprises interacted human genes affecting various diseases related cell signaling pathways correlating the use of aromatherapy. This database could be a useful resource to the plant’s growers/producers, an aroma/fragrance industrialist, health professionals, and researchers exploring the potential of essential oils and aroma compounds in the development of novel formulations against human diseases.

## Introduction

Both flowers and leaves of plants emit aroma compound that moved through the air and is detected by the olfactory system of animals. The complexity of aroma is still challenging because smell of any flower or plant is not due to a single chemical compound. Although plants and flowers consist of many chemical compounds, all of them do not contribute in the aroma. As the rose scent majorly influenced by major constituent compound (–) -*cis*-rose oxide and minor constituent compounds, namely, beta-damascenone (family – rose ketones) and beta-ionone of the plant’s essential oil, while other compounds like geraniol, nerol, (–) -citronellol, farnesol, and linalool contributions as minor.

Aroma is a mixture of volatile compounds with a molecular weight <300 and high vapor pressure, but the complete group of volatile compounds comprises thousands of inorganic and organic compounds stemming from major pathways of secondary metabolism (Frauendorfer and Schieberle, 2006). There are three significant pathways which involved in the biosynthesis process of the main aroma components in plants, such as the shikimic acid pathway by which eugenol (cloves) biosynthesized, degradation of lipids for the formation of short-chain alcohols and aldehydes and terpenoid pathway by which geraniol (Rose) and menthol (Peppermint) are synthesized. These aroma molecules act as semiochemical (mixture or chemical that carries a message for the purpose of communication), pheromones, defense mechanism, allow animals to recognize and detect individuals. The pheromone aroma molecules are essential for mating choices, sexual behavior, fertilization, and nursing and to warn kin in situations of danger (alarm pheromones) and to defend against predators. Aroma molecules are also involved in communication and interaction like plant–plant interaction (Das et al., 2013) and plant–animal interactions (Herrera and Pellmyr, 2002). These interactions and communications were performed through pollination (Schaefer et al., 2004), while another property of aroma molecule is a plant’s defense response against herbivores (Glaum and Kessler, 2017). Bacteria also emit a wealth of aroma molecules with an influence on plants, fungi, animals, and bacteria (Vet and Dicke, 1992; Heil and Silva Bueno, 2007). Aroma compounds can also be found in food, spices, perfumes, wine, fragrance oils, and essential oils. These molecules form biochemically during ripening of fruits and other crops. Aroma fragrance molecules are of great commercial interest, resulting in many applications of volatile aroma molecules in research, health, food, cosmetic, and health industries.

A comprehensive information of plants based aroma molecules (2D and 3D structures), aroma or fragrance type, essential oils, respective commercial plants varieties available for cultivation in India and abroad, therapeutic potential in terms of biological activities, physicochemical, stereo, and pharmacokinetics (ADMET) properties of aroma molecules, interacted human genes and corresponding essential oil’s export/import trade data trend information are not publicly available for both scientific research and industrial use. It was, therefore, our goal to develop a database AromaDb which cover basic and advance information of aroma molecules and provide a platform for comparative analysis and quantitative structure aroma relationship studies (QSAR).

The AromaDb database would be helpful to answers the queries of researchers, industries, and growers related to aroma compounds and essential oils. However, a variety of database resources of, essential oils, scent, fragrance and flavor (synthetic) components are already reported but still limited at some extent focused on certain subgroups, for example, SuperScent (Dunkel et al., 2009), OdorDB, Pherobase, EssOilDB (Kumari et al., 2014); ScentBase^1^, AroChemBase^2^, and Flavornet^3^ (**Table 1**). Consequently, these databases are good but useful for special purposes, and therefor there is a need for a comprehensive listing of commercially important volatile aroma molecules found in plant’s essential oils from different local and globally grown aromatic plants with information of chemotype-specific varieties, type of fragrance (aroma), physicochemical properties of aroma molecule, chemical identification, chemical structures (2D & 3D) for free downloads, Pharmacokinetic properties (ADMET), effect of aroma on human genes, their vapor pressure and logP,” and export and import trade data trends in terms of global demand and business turnover in India and abroad, so that to guide the future research directions for researchers, growers, farmers, and industries.

**Table 1:** Summary of existing databases related to aroma, odor, scent, essential oil, flavor, toxic compounds, and volatile molecules with its website addresses and descriptions.

S.No.	Database	Description	Website address
1.	SuperScent	A database focusses on scent molecules (including synthetic compounds) related to flavors and scents.	http://bioinf-applied.charite.de/superscent/
2.	OdorDB	A database focusses on a database of odor molecules related to the olfactory receptor database.	https://senselab.med.yale.edu/OdorDB/
3.	Pherobase	A database focusses on pheromones.	http://www.pherobase.com/
4.	EssOilDB	A database focusses on plant’s essential oils reflecting terpene composition, GC/MS data of essential oil, and variability in the plant kingdom.	http://nipgr.res.in/Essoildb/
5.	ScentBase	A database focusses on a compilation of floral scent components.	http://www2.dpes.gu.se/SCENTbase.html
6.	AroChemBase	A database focusses on compounds for aroma and chemical analysis using gas chromatography and Kovats index calculations.	http://www.alpha-mos.com/
7.	Flavornet	A database of volatile compounds found in the human olfactory perception space.	http://www.flavornet.org/flavornet.html
8.	SuperToxic	A comprehensive database of toxic compounds.	http://bioinformatics.charite.de/supertoxic
9.	mVOC	A database of microbial volatiles.	http://bioinformatics.charite.de/mvoc
10.	KNApSAcK Metabolite Ecology	A database covering information on the relationships between volatile organic compounds (VOCs) and their emitting organisms.	http://kanaya.naist.jp/MetaboliteEcology/top.jsp

## Materials and Methods

### Data Selection and Resources

Data were retrieved from the national and international literature and various web database resources or papers published in national and international journals. The database includes records from more than 100 scientific journals related to aroma (fragrance) and flavor. Following web resources were used to select the required data, such as selection of floral scents/fragrance based on medicinal and aromatic plants volatile (Baldwin et al., 2006) (**Table 2**). The abstracts were screened searched against chemical names and synonyms of chemical compounds on given literature search engines. The information regarding adverse effect or allergic responses (skin irritation toxicity) by some aroma molecules is covered in the AromaDb database under fields “physical and ADMET properties” which was calculated through TOPKAT module of Discovery Studio v3.5 software (Accelrys, San Diego, CA, United States). Compounds human genes interaction data was retrieved from the EPA (United States Environmental Protection Agency)^4^ and ACToR^5^ information portal by searching all compounds using CAS ID.

**Table 2:** Details of supporting databases and web resources used in AromaDb database development.

S.No.	Database description	Website address
1.	Natural products from Record of Natural Products (RNP)	http://www.acgpubs.org/RNP/
2.	Human genes interaction with essential oil compounds through Human Gene Database (Gene cards)	http://www.genecards.org/
3.	Clinical trials data from ClinicalTrials.gov	https://clinicaltrials.gov/ct2/home
4.	Aromatic plants molecules fragrance information retrieved from Food and Agriculture Organization of the United Nations (FAO-UN)	http://www.fao.org/home/en/
5.	Trade data of commercially important aromatic and medicinal crops from United Nations Commodity Trade Statistics Database (UN Comtrade)	http://comtrade.un.org/db/
6.	National trade data of plants essential oils through Department of Commerce, Ministry of Commerce & Industry (Govt. of India), India	http://commerce.gov.in/
7.	PubChem compounds structures (2D and 3D)	http://pubchem.ncbi.nlm.nih.gov/
8.	PubMed literature search database	www.ncbi.nlm.nih.gov/pubmed
9.	Google Scholar literature search database	https://scholar.google.co.in/
10.	TOPKAT module of Discovery Studio v3.5 molecular modeling software (Accelrys, San Diego, CA, United States)	http://accelrys.com/
11.	CSIR-CIMAP Annual Reports (2009–2015), India	http://www.cimap.res.in/english/index.php/annual-report
12.	Improved Varieties – Medicinal and Aromatic Plants and JMAPs data, CSIR-CIMAP, India	http://intranet.cimap.res.in/cimvariety/

### Database Development Backend Information

**FIGURE 1:**
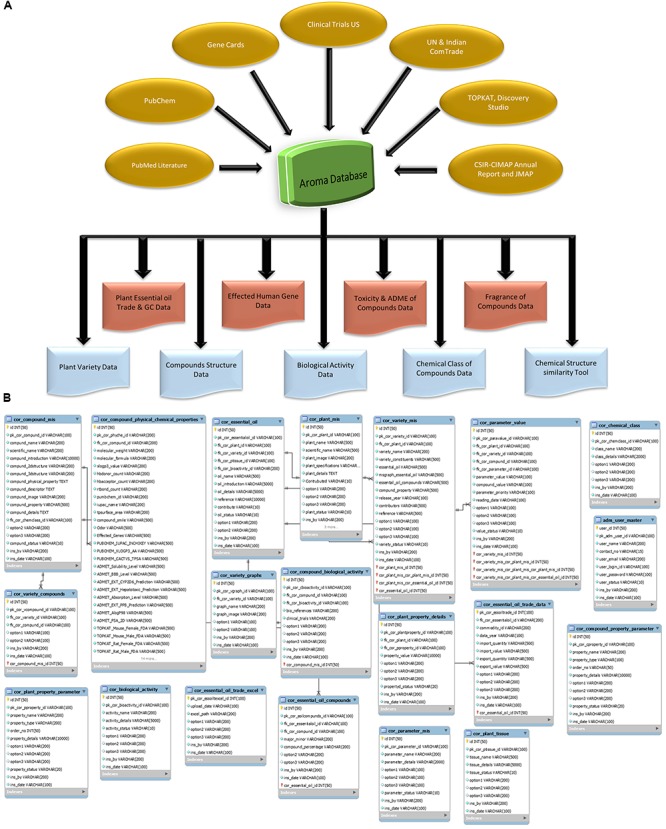
Details of the AromaDb database **(A)** architecture, and **(B)** entity-relationship diagram.

AromaDb database is developed on Apache HTTP server, which is platform independent and available as open-source software. The database is developed on MySQL for storing the information in the backend. The database website front end is developed in PHP, HTML, CSS, and JavaScript. AromaDb comprises basic and advances molecular information retrieved from different resources as shown in **Figures 1A,B**.

### Structure Similarity Search Tool Application and Structure Editor

Structure editor and structure similarity search tools in AromaDb for structural similarity within the database and from the extended database, i.e., ChemAxon SMILES based search option was used, for displaying 3D structures, JSmol was used which is available as open-source JavaScript viewer for chemical structures in 3D^6^. JSmol is the extension of the Java-based molecular visualization applet Jmol^7^ as an HTML5 JavaScript-only web app. Similarly, JSME (JavaScript Molecular Editor) was applied for the built-in molecule editor, which allows the user to screen with self-edited molecules. JSME is a free molecule editor written in JavaScript^8^.

## Results

### Database Content

The major parameters in the database were plants, variety, essential oils, chemical molecules, chemical group, trade data, structural data, and biological activity of essential oil, compounds and their interacting genes, etc. For better performance, all the data is kept distributed in several interrelated tables logically. For wide complex searching search engine was used to check each link of the database website and accordingly showed the matched results (**Figure 1B**). All the information in AromaDb database can be categorized into three categories: primary, secondary, and tertiary information. The database information was manually curated and selected from various sources such as published international literature, CSIR-CIMAP, Lucknow^9^ essential oils monograph, web link annual reports, journal (JMAPS; *Journal of Medicinal and Aromatic Plant Sciences*; Baldwin et al., 2006), newsletters, and books. The primary information has been retrieved from the literature, these information’s consists of the following major fields namely: (i) plant details (ii) essential oil name, and (iii) plant variety.

**FIGURE 2:**
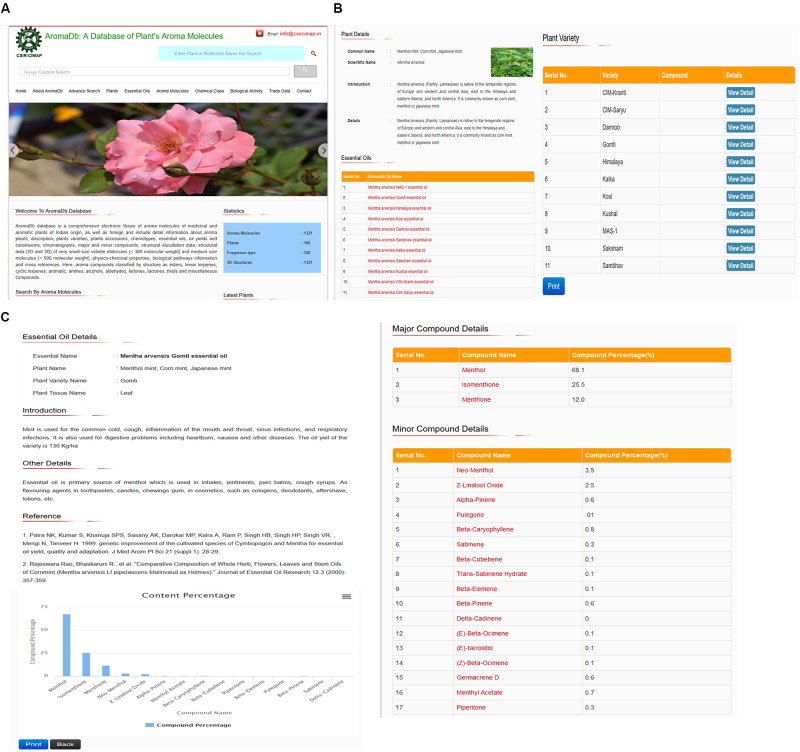
Snapshots showing details of the AromaDbdatabase **(A)** homepage, **(B)** Indian plant varieties, and **(C)** essential oil constituents.

The secondary information, which was derived from the plant essential oil (primary information) includes the essential oil description details, chemical constituents, major minor compound details, content percentage, and export–import essential oil trade data. The tertiary information is further derived from secondary information which comprises detailed information about the essential oil compounds as followed: IUPAC name, Chemical class biochemical classes (e.g., terpene hydrocarbons and oxygenated compounds: phenols, alcohols, aldehydes, ketones, esters, lactones, coumarins, ethers, and oxides), Fragrance type, Physical and chemical properties, Absorption and Metabolism information, Toxicological information, Ecological information, Hazards information, and compound bioactivity data for therapeutic use or drug formulation development. This information provides the user a comprehensive aroma molecules database, together with substantial options, such as chemical compounds search based on structural similarity using compound Canonical SMILES within databases. **Figure 2** summarizes snapshots of database home page (**Figure 2A**), plant and varieties details (**Figure 2B**), and essential oil details (**Figure 2C**).

### Results of Structure Editor and Structure Similarity Search Tools

The AromaDb database provides the diverse and commercially important aroma molecules, fragrance types, essential oils, aromatic plants, trade data, and other industrially important information as required while formulating any herbal product formulation for human uses. We have incorporated various tools for making the database easy and more convenient for users. The database contents could be accessible with different search tools, options, e.g., simple and advance search tools. The simple search tool is powered by Google search engine, which search data from within the AromaDb database and also from public databases, where another mining tool search the query within the database, the Advance search tool option further divided into two types: (i) search based on structural similarity (within AromaDb database) and (ii) search based on structural physicochemical and pharmacokinetics (ADMET) properties. The database search fields enable the user to look for compounds using physicochemical properties, plants, varieties, chemotype, essential oil, aroma molecules, chemical classification, biological activity, etc. Database allows the user to choose certain functional groups, species or range of molecular weights, which search whole entries and retrieve the user required results. To mine the database user require a 2D molecular structure or structure canonical SMILES code (Simplified Molecular Input Line Entry System) or a MOL-file of interested aroma compounds. Structure drawing/editing option is provided in the AromaDb database with the help of JSME (JavaScript Molecular Editor; JSME Homepage^10^). With the help of JSME user can draw either full structure or part of it. The most similar database entries are listed in the order of structural similarity. For each compound, details of plant, plant name, variety, essential oil constituents, chemical class, ADMET properties, 2D/3D chemical structures, trade data, and the SMILES based similarity search percentage are presented. Furthermore, a similarity search to find the most similar compounds analogs are provided. Additionally, similar information can be viewed separately by accessing database header fields or the menu.

### Browsing Information in AromaDb

**FIGURE 3:**
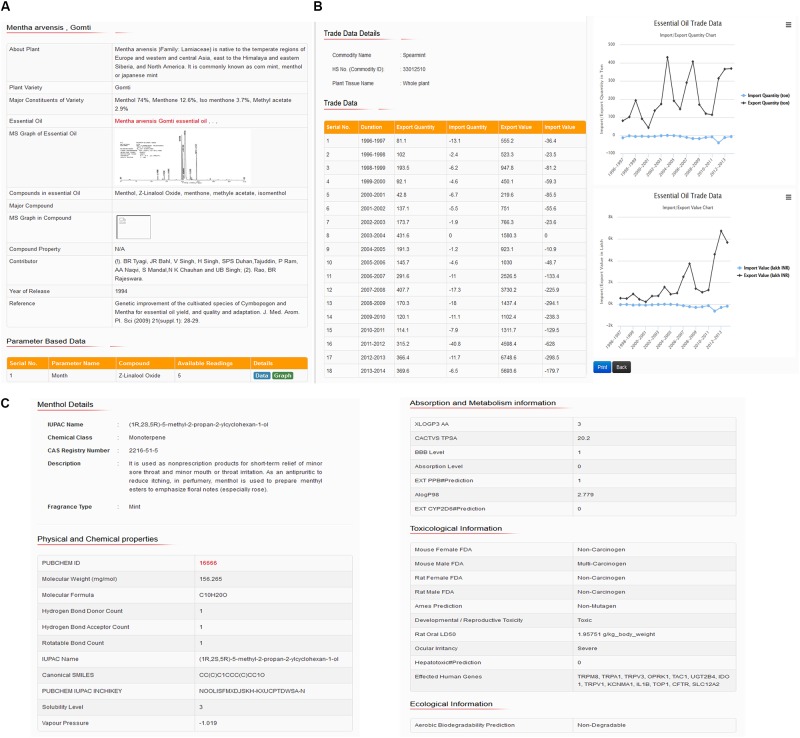
Snapshots showing **(A)** constituents, GC–MS data and month’s wise variations in oil yield, **(B)** essential oil trade data and comparative graphical plots, and **(C)** aroma compound description and 3D structures for downloads.

The user can find IUPAC name, synonyms or common names, CAS registry number, chemical classification, functional groups, molecular weight, pharmacokinetics data, and commercially cultivated aromatic plants and their varieties in which the aroma compounds have been found, together with the authentic references and bibliography, as well as in-house published contributed data (CSIR-CIMAP, Lucknow, India^11^). Furthermore, the aroma compounds were classified according to their structures, chemical features, quality of fragrance (aroma type), essential oil, yield, major and minor constituents, (Gas Chromatography-Mass Spectrometry) GC–MS spectra, last 18 years trade data (1996–2014) of the most commercially used 34 aromatic plants in India and other industrial research purpose information. **Figure 3** represents the snapshots of chemical constituents of essential oil, GC–MS analysis data and months wise variations in oil yield (**Figure 3A**), essential oil trade data and comparative graphical plots (**Figure 3B**), and aroma molecule description, a 3D structure for downloads (**Figure 3C**).

### Information About Putative Therapeutic Free Human Targets or Genes in AromaDb

The other useful feature of the AromaDb database is the information of aroma molecules and related interacting human therapeutic genes or proteins involved to modulate the biological system biological pathways and for unraveling the underlying mechanism of action and therapeutic potential of these aroma molecules along with supporting references. Also enlisted the therapeutic important aroma molecules along with the option to see the related aromatic plants, essential oils, export and import trade data, and other information.

### Display of Essential Oil Trade Data Information

The graphical analysis of trade data suggests the consumption and demand data trend in India and the world or own country (India) as a guideline or forecasting tool to the aroma or essential oil industries and growers (farmers or producers) to streamline their resources, economy, and time according to expected demand of respective essential oils in the world and therefore indirectly helps to improve the socio-economical condition of farmers and producers. Here, the database entries have been clustered according to the quality of their aroma properties. A manually verified upload option through email allows the scientific community to contribute to the database. Here, the user can import a MOL-file together with corresponding information of the compound. The AromaDb database will be updated on a quarterly basis.

### Comparative Display of Physical Properties and Safety Prediction Data

The database features the properties related to aroma molecule, hazard identification, exposure controls and personal protection, physical and chemical properties, toxicological and ecological information. Compound identification properties include chemical name, IUPAC name and chemical class. Hazards identification includes properties related to physical hazards, health hazards such as skin corrosion and ocular irritancy property. Toxicological properties such as irritation, absorption level, aqueous solubility level, LogP (octanol/water partition coefficient), polar surface area (PSA), and blood–brain barrier level indicate the precautionary properties related to exposure controls and personal protection (eye/face protection, skin protection, respiratory protection, and thermal hazards). Physical and chemical properties indicated by molecular weight, molecular formula, fragrance type, LogP (*n*-octanol/water), H-bond donor and H-bond acceptor, rotatable bond, topological polar surface area (TPSA), IUPAC name, InChIKey, aqueous solubility level, vapor pressure, PubChem (NCBI, United States) (Wang et al., 2009) database ID and SMILES information. Toxicological information includes properties related to information on likely routes of exposure such as inhalation, skin contact, eye contact, ingestion, symptoms related to the physical, chemical and toxicological characteristics, information on toxicological effects, e.g., acute toxicity (LD_50_ – dermal/oral, rabbit/rat, mg/kg), skin corrosion/irritation, serious eye damage/eye irritation, respiratory or skin sensitization, germ cell mutagenicity (genotoxic), carcinogenicity, reproductive toxicity, specific target organ toxicity (single exposure/repeated exposure), and aspiration hazard. However, the ecological information includes properties related to eco-toxicity (environmentally hazardous), persistence, and degradability. Since these aroma molecules used in aroma based natural therapy (aromatherapy) in traditional herbal medicine system of India (Ayurveda and Unani) and other East Asian countries, therefore, database covered properties related to pharmacokinetics such as absorption, distribution, metabolism, excretion, and toxicity (ADMET). These properties indicate drug-likeness properties, ADME compliance, and toxicity risk assessment data. Moreover, the database also includes information related to the molecular interaction of aroma molecules/essential oils with human proteins (genes) directly or indirectly affecting the metabolic processes and therefore causing their useful biological activity or responses. This information is supported with cross-references or proper evidence based on reported publications and therefore offer a possibility for biological interpretation of these aroma molecules. Moreover, an investigation of medical effects is also possible by browsing the “biological activity” field for each essential oils/aroma molecules. The comparative trends or pattern plots shows the emitted aroma compounds of the chosen species compared with all other plants species, which emit these compounds. The comparative data analysis through graph plots shows the unique pattern of aroma molecules (fingerprints), essential oil, yield, major constituent percentage (chemotype), trade analysis trend, and others based on changes in different agro-morphological parameters, e.g., soil type, stress conditions, temperature, weather type, months wise oil yield, etc., which are important feature for distinguishing between more or less useful features/parameters during agriculture practices and/or cultivation by farmers, or industrial growers. As an example, a snapshot represents these calculated properties of aroma showing molecule, chemical identification, description and 3D structure for download snapshot represented in the given figure (**Figure 3C**).

## Discussion

It was difficult to explore such information about plants, its variety, essential oil constituents, and essential oil trade data, on a single platform in addition to the aroma molecules information about its physicochemical properties, absorption and metabolism information, toxicological information, ecological information, Hazards identification, and compound biological activity with its fragrance and interacting human genes information.

### Browsing and Searching Database Contents

Database contents can be seen in two ways: (i) browsing different data fields available on the header menu on the home page such as, plants, essential oils, aroma molecules, chemical classes of aroma molecules, biological activity, and trade data, and (ii) searching database contents through advanced search option and wild search through Google’s custom search option. Users can search any text within the database based on text similarity concept through Google’s custom search option (wild search or complex search) available in the header of the database. A similar analysis with the help of representing snapshots of the database showing database home page search parameters (**Figure 2A**), plant and varieties details (**Figure 2B**), and essential oil details (**Figure 2C**).

### Properties Search by Entering Values

Besides, users can also limit searches using ranges of physiochemical data, e.g., maximum and minimum values of TPSA (Topological Polar Surface Area) (**Figures 3A–C**). Database snapshots of these properties and parameters showing chemical constituents of essential oil, GC–MS data and months wise variations in oil yield (**Figure 3A**), essential oil trade data and comparative graphical plots (**Figure 3B**), and aroma molecule description and 3D structure for downloads (**Figure 3C**).

### Similar Structures Search and Downloading of Aroma Molecules

**FIGURE 4:**
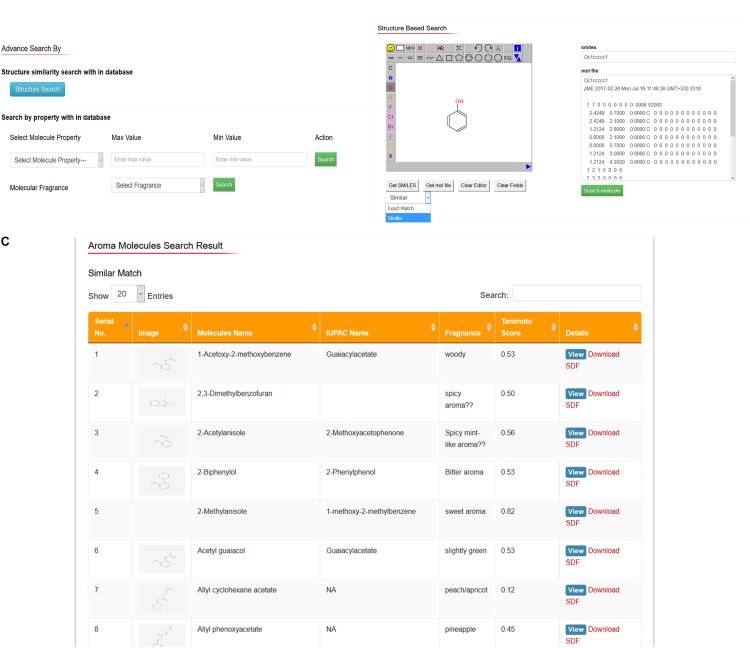
AromaDb database snapshots showing **(A)** advance search options based on different properties, fragrance type and toxicological parameters of aroma molecule, **(B)** drawing tool for structure-based search, and SMILES or MOL file basis search options, and **(C)** results of structure-based search based on SMILES similarity.

Users can search and download interested aroma molecules (2D and 3D chemical structures) through structure search option. In this option, users can draw or edit any structure of their interest and convert it to SMILES and MOL file format and subsequently search whole AromaDb database aroma molecules and resulted most similar matched structures available in the database and can easily see details of these matched small molecules or free downloads the structures. For example, if the user draws the phenyl ring in the JME editor and enter the option either “get SMILES” or get MOL file’ resulted in SMILES or MOL file data would be shown on the other side of JME editor and subsequently enter the key similarity search within database, results of matched structures showed in the new web page, with no exact match aroma molecule found, and enlisted the similar matched compounds, e.g., (*E*)-cinnamyl acetate, 1,1-diisobutoxy-2-phenylethane, 1,1-dimethoxy-2-benzylideneheptane, etc. All these matched structures have phenyl ring in common. Moreover, the user can see further details of each matched compound by entering key on “details” button. Details of aroma compound include calculated properties related to aroma molecule identification, hazards, physical and chemical properties, pharmacokinetic properties, toxicological information, and ecological information. The database also represents the 2D structure, 3D structure visualization in 3D structure viewer window with spin-on/off option so that to see the structural conformations quickly with free download option. The compounds search outputs are oriented to explore the matched compounds by following the “details” hyperlink to the table of physical, ADMET and safety properties described earlier. **Figure 4** showing snapshots of advanced search options based on different properties, fragrance type and toxicological parameters of aroma molecule (**Figure 4A**), drawing tool for structure-based search option as well as SMILES or MOL file basis (**Figure 4B**), and results of structure-based search based on SMILES basis structural similarity (exact match and similar hits; **Figure 4C**).

### Search by Selecting Data Fields

Similarly, naive users can directly see and select the interesting data through search menu buttons provided at the end of the home page of aroma database and the further user may browse more information without wasting time on thinking about aroma (fragrance), aroma molecules name, essential oils, plant varieties, and plants. This type of search display option will help the fresh researchers, scholars, and students. For example, a new user can directly browse the home page header menu field “Plants,” which will display the list of all aromatic plants with their common and scientific names and a number of their available plant varieties or highly yielding chemotypes. Users can see more details of these plants by clicking on the button “View Detail.” For example, if the user wishes to search for Menthol mint plant (*Mentha arvensis*), which included at present 11 mint plant varieties or chemotypes based on different aroma molecules constituent’s ratio variations. Users can see more details such as brief introduction, family, localization, uses, essential oils types, and the name of 11 mint varieties used by Indian growers or farmers. Users have the option to print or save this data. In this page, users have two options to see further details; (i) essential oil types and (ii) variety details. For example, if the user searches for *M. arvensis* MAS-1 essential oil details, database will display brief introduction, major constituent Menthol with 84% with multiple minor components such as Menthone (5.8%), etc., comparative graph plot showing percentage ratio of different chemical constituents of *M. arvensis* MAS-1 essential oil (**Figure 2C**), and the Indian scientists or researchers contribution, if any, showed in the references. Users have the option to print (or save) this data and graph plot. Also, the user can directly move to see details of major or minor compounds and yield percentage by simply enter on the name of compounds.

Moreover, if the user enters on *M. arvensis* variety Gomti button, the database will display a tabular text data showing brief details of plant, variety name, major constituents of given variety such as Menthol 74% in ratio, menthone 12.6%, isomenthone 3.7%, and methyl acetate 2.9%. Beside this database show details of essential oil, GC–MS graph (if available) of essential oil, major compounds peak in GC data, compound property, year of plant variety release and complete reference (**Figures 3A–C**).

### Agronomic Parameters Based Gaphical Data Analysis

At the same time, the database also covers data related to different agronomic parameters based and provide an option to the user to see either in tabular or graphical forms. For example, in the case of *M. arvensis* Gomti variety, the database covers five observation readings revealing variations in Z-Linalool oxide compound percentage ranges from 2.2 to 2.7% from January to May 2015. This data suggest that the highest yield of Z-Linalool oxide was 2.7 February. Likewise, the user can see other parameter based graph plot analysis and get benefited from future cultivation, for example, if the user sees the Himalaya variety of *M. arvensis*, there are five readings based on the age of the plant (in days) and menthol compound yield percentage. This data showed that the study was performed for 30 days old plants to 150 days old plant (menthol mint) and menthol yield ranges from 70.66 to 82.18%. Graphical analysis revealed that highest yield obtained at 120 days old plant (**Figure 2**). A similar analysis with the help of representing snapshots of the database showing database home page search parameters (**Figure 2A**), plant and varieties details (**Figure 2B**), and essential oil details (**Figure 2C**).

## Conclusion

The AromaDb database is a useful tool to retrieve information about aroma molecules, aroma or fragrance types, essential oils, plants varieties, bioactivity of essential oils or aroma molecules, toxicological and ecological data, and trade data. The database provides, 3D structures of aroma compounds for free downloads and option to see the essential oil yields or constituents percentage variation trends at different agro-morphological conditions during plant growth. The included data on aroma molecules along with a focus on associated plants and their essential oils chemotype (varieties) will enable systematic experimental approaches on the relation between structural similarities, essential oils, and aroma (fragrance) type and aroma chemical classes. Besides, last 18 years global export and import trade data of plants essential oils will educate the growers or farmers to prioritize the cultivation of aromatic plants based on expected global demand. Furthermore, structure comparisons of self-edited molecules with the database aroma molecules as well as the external database may allow a first rough estimation of the potential aroma of new chemicals. The AromaDb database is a free resource with embedded screening functions for aroma molecule based on molecular weight, plants, varieties, essential oils, fragrance or aroma type, toxicological and ecological information (allergic or toxic responses).

## Availability and Implementation

The database is available at URL: http://bioinfo.cimap.res.in/aromadb/.

## Author Contributions

YK carried out the database entry, data searching and retrieval, download, designed 2D and 3D structures, participated in the data analysis, and manuscript writing. OP developed the preliminary database offline using PHP and MySQL for initial data entry. HT add the trade data of essential oil based plants commodities and economic profile in Indian currency. ST provided 25 aroma molecules to the database and other properties. MG also provided small molecules and corrected chemical classes of aroma molecules and corrected the chemistry part. L-UR provided data related to Indian plants varieties, essential oils details, constituents, and contributed in the design of the database. RL guided to add published in-house data related to aroma molecules, essential oils, aroma plants, and bioactivity. MS coordinated in database web hosting and IT support. FK conceived the study, contributed to its design, ER relationship, and drafted the manuscript. MD Addition of updated bioactivity (*in vitro*) data for essential oil/aroma molecules with their cros references. All authors read and approved the final manuscript.

## Conflict of Interest Statement

The authors declare that the research was conducted in the absence of any commercial or financial relationships that could be construed as a potential conflict of interest.

## Footnotes

1 https://scentbase.com/

2 www.alpha-mos.com

3 http://www.flavornet.org/

4 https://www.epa.gov/

5 https://actor.epa.gov/actor/home.xhtml

6 http://sourceforge.net/projects/jsmol/

7 http://jmol.sourceforge.net/

8 http://peter-ertl.com/jsme/

9 www.cimap.res.in

10 http://peter-ertl.com/jsme/

11 www.cimap.res.in
